# Sublingual Delivery of Astaxanthin through a Novel Ascorbyl Palmitate-Based Nanoemulsion: Preliminary Data

**DOI:** 10.3390/md17090508

**Published:** 2019-08-29

**Authors:** Andrea Fratter, Damiano Biagi, Arrigo F. G. Cicero

**Affiliations:** 1 Research and Innovation Technology, Nutraceutical Department, Labomar Research, 31036 Istrana, Italy; 2 Medical and Surgical Sciences Department, University of Bologna, 40138 Bologna, Italy

**Keywords:** astaxanthin, nanoemulsion, sublingual delivery, ascorbyl palmitate, Franz cell

## Abstract

Astaxanthin is a carotenoid extracted from several seaweeds with ascertained therapeutic activity. With specific reference, astaxanthin is widely used in clinical practice to improve ocular tissue health and skin protection from UV ray damages. Despite its well-documented pleiotropic actions and demonstrated clinical efficacy, its bioavailability in humans is low and limited because of its hydrophobicity and poor dissolution in enteric fluids. Furthermore, astaxanthin is very unstable molecule and very sensitive to light exposure and thermal stress. Taken together, these pharmacological and chemical–physical features strongly limit pharmaceutical and nutraceutical development of astaxanthin-based products and as a consequence its full clinical usage. This work describes the preliminary in vitro investigation of sublingual absorption of astaxanthin through a novel ascorbyl palmitate (ASP) based nanoemulsion.

## 1. Introduction

**Figure 1:**
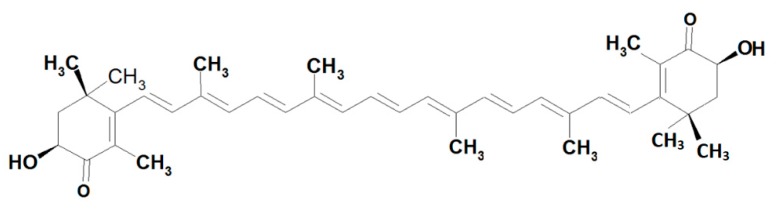
Structure of Astaxanthin.

Astaxanthin (C40H52O4;6S)-6-Hydroxy-3 [(1E,3E,5E,7E,9E,11E,13E,15E,17E)-18-[(4S)-4-hydroxy-2,6,6-trimethyl-3-oxo-1-cyclohexenyl]-3,7,12,16-tetramethyloctadeca-1,3,5,7,9,11,13,15,17-nonaenyl]-2,4,4-trimethyl-1-cyclohex-2-enone) (Figure 1) is a lipid-soluble xanthophyll keto-carotenoid with molecular mass 596.841 g/mol [1].

It is the responsible of the red color of some crustaceous and fishes [2]. The most known pharmacological activity of astaxanthin is the antioxidant one [3] but, contrary to other carotenoids, [4] it seems to also exert direct anti-inflammatory activity and to activate Peroxisome Proliferator-Activated Receptors [5].

In (usually small and short-term) clinical trials, oral supplementation with astaxanthin not associated with other nutraceuticals has demonstrated to be protective against UV-induced skin deterioration and helps maintain healthy skin in healthy people [6], to improve liver parameters in climacteric women [7], to protect the vocal fold from injury and inflammation caused by vocal loading [8], to increase the choroidal blood flow velocity in healthy subjects [9], to reduce LDL-cholesterolemia and oxidative stress in overweight patients [10,11], to increase HDL-cholesterolemia and serum adiponectin levels in mildly dyslipidaemic subjects, [12] to prevent oxidative damage in smokers by suppressing lipid peroxidation and stimulating the activity of the antioxidant system [13], and to improve symptoms in patients affected by functional dyspepsia (especially if infected by *Helicobacter pylori*) [14]. 

In some trials, supplementation with astaxanthin was also shown to prevent and reduce oxidative stress in young soccer players, [15,16] but not in well-trained cyclists [17]. In a previous study, it improved performance in cyclists, [18] while in a recent study it does not augment fat use or improve endurance performance [19]. However, a recent randomized controlled clinical trial showed no effect of astaxanthin on arterial stiffness, oxidative stress, or inflammation in renal transplant recipients [20].

The apparent contrast between positive and neutral effects observed in clinical trials are mainly related to the different dosage used, but also to the largely different bioaccessibility of the tested pharmaceutical forms [21].

It is well ascertained, indeed, that astaxanthin is poorly bioavailable in humans [22] from the conventional pharmaceutical forms, particularly because of its high lipophilicity that precludes the overall enteric bioaccessibility and because it can be enhanced by modified lipids and surfactants capable of making it more hydro-dispersible [23].

Many attempts have been dedicated to projecting pharmaceutical forms with the aim of improving bioaccessibility and overall bioavailability of astaxanthin and in this frame, nanoemulsions seem to play a pivotal role according to numerous published papers [24,25,26]. 

Given the potential interest of developing more effective forms of astaxanthin supplements, the aim of our study was to evaluate, likely for the first time, a novel liquid nanoemulsion to promote astaxanthin sublingual delivery by means of an in vitro model assessing its permeation through porcine lingual specimens. 

## 2. Materials and Methods

### 2.1. Materials

A Franz cells device was purchased from Copley Scientific (Nottingham, UK), surgical blades from Tekno Optik-Chirurgie GmbH (Tuttlingen, Germany), and scalpel handle from Moretti Spa (Cavriglia, Arezzo, Italy). Malvern Zetasizer Nano series (DLS device) was purchased from Malvern Panalytical (Mlavern, UK). A HPLC-DAD device was purchased from Perkin-Elmer (Series 200, diode array, Waltham, MA, USA). A mechanical stirrer (LG series) and heating plate (RC series) were purchased from Velp Scientifica (Usmate, MB, Italy). Astaxanthin (Astapure^TM^ 10% titration in astaxanthin) was purchased from AlgaTech (New York, NY, USA), astaxanthin standard analytic (>97% from *Hematococcus pluvialis*) was purchased from Sigma-Aldrich (Milan, Italy), physiological solutions were purchased from BS Medital Spa (Grosotto, SO, Italy). Polysorbate 80 (Veremul T 80) was purchased from Veronelli SPA, Milan, Italy; ascorbyl palmitate was purchased from ACEF, Fiorenzuola, Piacenza, Italy; caprylic/capric triglycerides (Labrafac Lipophile WL 1349) were purchased from Gattefossè, Milan, Italy; deionized water was obtained from inverse osmosis industrial device. 

### 2.2. Preparation of Porcine Sublingual Epithelium

For the experiments, fresh pork tongue was used. Pig’s tongue was obtained from a 6-month-old pig, weighing around 80 kg. The tongue was withdrawn in a local slaughterhouse, transported to the laboratory under vacuum, and immediately used (within 2 h). 

**Figure 2:**
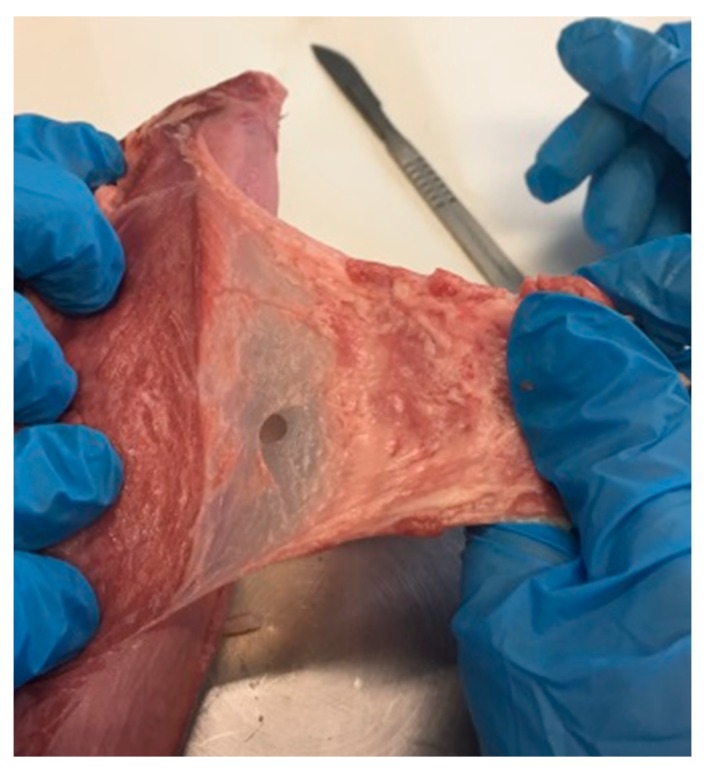
Sectioning of the epithelium from pig tongue.

Initially, the tongue was placed in physiological solution for about ten minutes, then washed out with new physiologic solution three times, and then sectioned to get the epithelium specimens. The sublingual (ventral) epithelium was sectioned using a scalpel to separate it from underlying connective tissue (Figure 2). 

**Figure 3:**
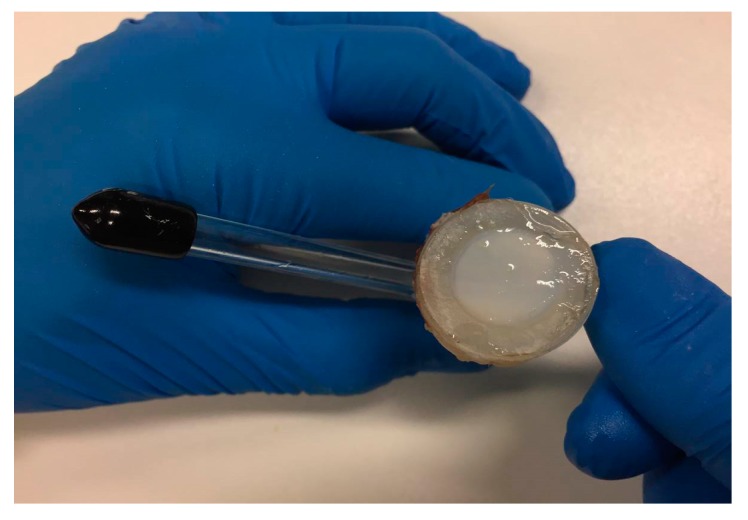
Epithelium specimen placed over the Franz cell chamber.

Before clamping the epithelium into the Franz cell chamber (Figure 3), it was washed with PBS 10× pH 6.6–7.4 three times [27,28].

In order to ascertain the correct device assembly and the porcine lingual epithelium integrity, the donor compartment was filled with physiologic solution, to be sure that no liquid overcame the membrane reaching the receptor compartment, meaning that no lesion occurred in the membrane and that the cell was well assembled.

### 2.3. Preparation of the Astaxanthin Containing Nanoemulsion

Astaxanthin nanoemulsion was prepared using the components listed in Table 1.

**Figure 4:**
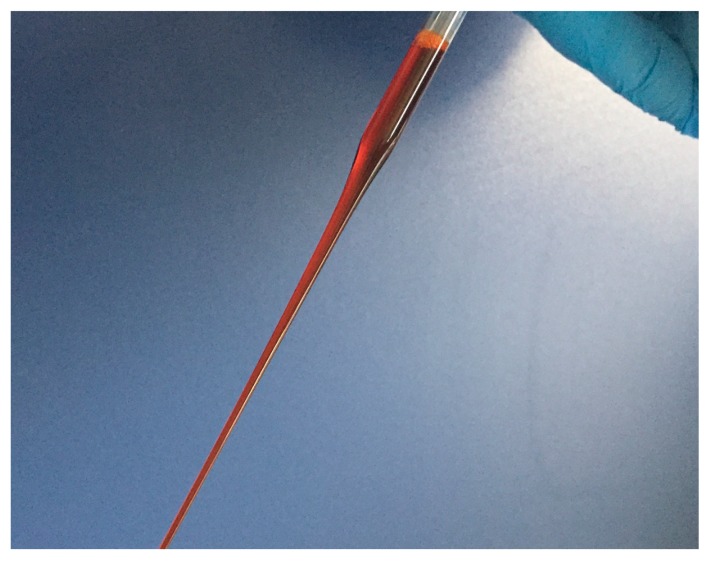
Astaxanthin containing nanoemulsion. The system appears perfectly clear with an intense red color conferred by the carotenoid entrapped.

Emulsion was prepared incorporating Astapure^TM^ (10% *w*/*w* in astaxanthin) in the oily phase (equal to 0.15% *w*/*w* theoretic value of astaxanthin on the total nanoemulsion) composed of caprylic/capric triglyceride, and polysorbate 80 (PS 80) and ascorbyl palmitate, as the main high hydrophilic–lipophilic balance emulsifying agent and co-emulsifying agent, respectively. The preparation of the oily phase and the further steps to achieve the nanoemulsion were carried out in a dark room to protect astaxanthin from UV rays. Both the oily and water phases were warmed up at 50 °C and the final emulsification process was carried out at this temperature, slowly pouring the water phase into the oily phase under high-speed mechanical stirring. Soon after, the system was cooled down by placing the beaker containing nanoemulsion, appearing as a perfectly clear system with an intense red color (Figure 4), in an ice-water bath, maintaining low-speed stirring until the room temperature was reached, according to low energy PIT method [29,30,31]. 

### 2.4. Dimensional Characterization of Astaxanthin Containing Nanoemulsion

Samples of nanoemulsion containing astaxanthin were analyzed in triplicate with Dynamic Back Scattering device (DLS) to assess the average dimensional size of the oily droplets. 

### 2.5. Permeation Experiments

The incisions were made starting from the ventral part of the swine tongue, with particular attention given to removing only the outermost layer which, once cut, is completely transparent. Once the external epithelial tissue was sectioned, it was gently positioned in the appropriate space between the donor and the receiving chamber. A total amount of 1.26 g of the astaxanthin-containing nanoemulsion (equal to total 0.189 g of astaxanthin) was inserted from the upper apex, in the donor compartment of the Franz cell and placed at 37 °C under magnetic agitation (210 rpm). The acceptor chamber was filled up with degassed solution of PBS 10× (10 mL). The cells, before the start of the experiments, were allowed to equilibrate for 60 min in a water bath at 37 °C (according to Franz-Montan 2016) [26].

A stirrer was necessary to keep the system in a continuous flow to carry out solution withdrawals in which the active permeation is homogeneously dispersed. The samples were withdrawn after 15’, 30’, 60’, 2 h, 4 h. Samples (1 mL) were withdrawn from the receiving chamber for the HPLC analysis and the volume was replaced with the same amount of fresh buffer PBS, taking account of dilution effects.

The data obtained from the HPLC analysis relating to the title of astaxanthin in the receptor chamber were converted into mass per unit of surface (µg/cm^2^) of the permeating membrane. The surface area of the porcine lingual epithelium inserted in the chamber was calculated, starting from the chamber diameter, according to Equation (1):

(1) $$A=\frac{\left({\pi d}^{2}\right)}{4}{\mathrm{cm}}^{2}$$

where A is the surface of the chamber and *d* is the diameter of the chamber.

From the linear correlation obtained by relating the astaxanthin content in the donor chamber with time, the slope of the linear tract of the plot (*s*) was calculated: This data permitted the calculation of the apparent permeability coefficient (*P_e_*) through the following relation (Equation (2)) derived from the first Fick’s equation considering *C_d_* > *C_a_*:

(2) $$P_{e}=\frac{dC_{a}}{\mathrm{dt}}\times \frac{1}{A}\times \frac{V_{a}}{C_{d}}$$

where d*C_a_*/d*t* is the slope (*s*) of the linear correlation between the change in concentration of permeated astaxanthin in the infinitesimal time change, A is the permeation surface, *C_d_* is the concentration on the donor compartment, and *V_a_* is the volume of the acceptor chamber [32]. Once the permeability coefficient (*P_e_*) was calculated, according to Bortolotti F. et al. (2009), the flux at the steady state (*J_ss_*) was also calculated through the following Equation (3):

(3) $$J_{ss}=P_{e}\times C_{d}$$

where *J_ss_* is the flux at the steady state, *P_e_* is the permeability coefficient, and *C_d_* is the concentration of astaxanthin in the donor chamber [33].

Experiments were realized in triplicate (*n* = 3) and mean value of astaxanthin concentration (±SEM) permeated in the receptor liquid, at any time of withdrawal, was used to calculate the concentration of astaxanthin per cm^2^ (±SEM), flux (*J_ss_*) (±SEM), and apparent permeability coefficient (*P_e_*) (±SEM). Statistical elaboration of the data collected was realized through software SPSS according to T-Student method (*p* < 0.05). 

### 2.6. Titration of Astaxanthin in Raw Material, Nanoemulsion, and Permeation Specimens

Firstly, 100 mg of Astapure^TM^ (10% astaxanthin containing oil, raw material employed to fabricate nanoemulsion) was solubilized in 100 mL of acetone. Then, 500 mg of nanoemulsion containing astaxanthin was weighted in analytical balance and then solubilized in 100 mL of acetone. After that, the samples were sonicated for 15 min and then centrifuged and placed in vials. The samples were analyzed through High-Performance Liquid Chromatography with Diode-Array Detection coupled with UV-analyzer (HPLC-DAD-UV), DAD scan range was from 200 to 800 nm, with stationary phase composed of YMC Carotenoid column 4.6 mm I.D.× 250 mm (C30 bonded silica, Particle size: 5 μm, usable pH range: 2.0–7.5): YMC Carotenoid stationary phase provides sufficient phase thickness to enhance interaction with long chained molecules, therefore, geometric and positional isomers of conjugated double bonding systems, typical of carotenoids and their esters, are recognized and resolved [34]. Mobile phase composed of methylterbutil ether (MTBE)/methanol 90:10 and methanol. The flux was set at 1.3 mL/min and the wave length set at 470 nm. The same method was applied to assess titration of astaxanthin from the permeation specimens. Thanks to this procedure, encapsulation efficiency E*_e_* of the fresh fabricated nanoemulsion was calculated according to Equation (4) [35]:

(4) $$E_{e}\;\left(\%\right)=C_{Ast}\times 100$$

where E*_e_* is the encapsulation efficiency and C*_Ast_* is the concentration of astaxanthin loaded in the nanoemulsion during fabrication (time 0).

The titration results were expressed as the mean and standard error of the mean (±SEM) for each variable studied.

## 3. Results and Discussion

### 3.1. Dimensional Characterization of Astaxanthin Containing Nanoemulsion

**Figure 5:**
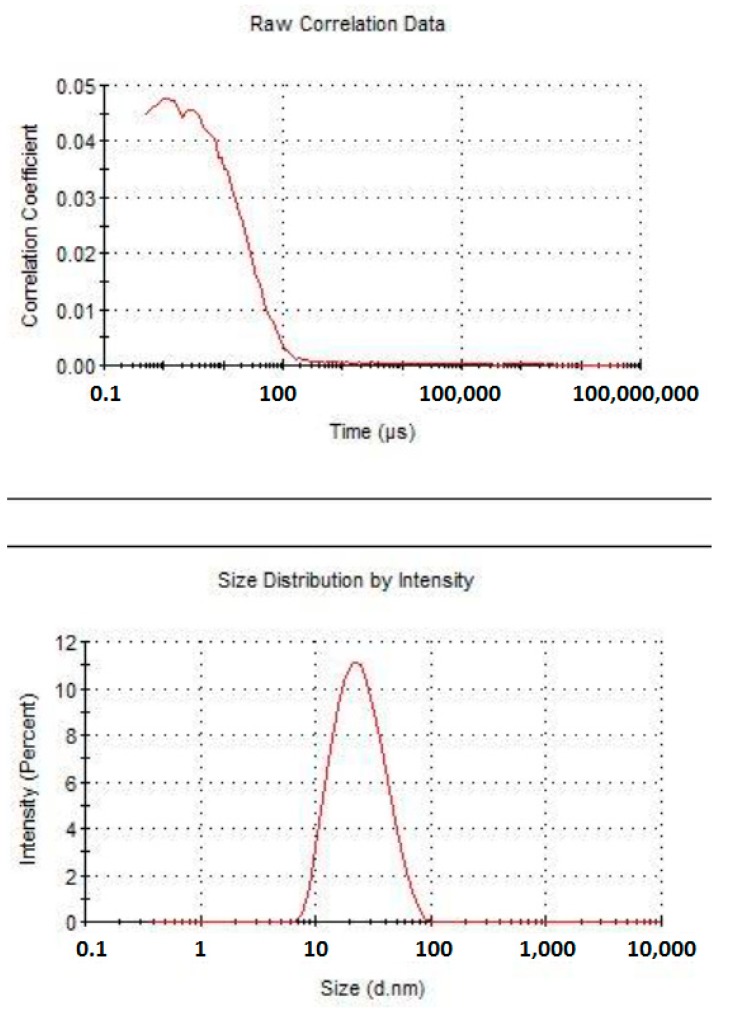
Graph plotting the correlation coefficient of astaxanthin nanoemulsion over time (**upper**) and size distribution by intensity (**lower**).

**Figure 6:**
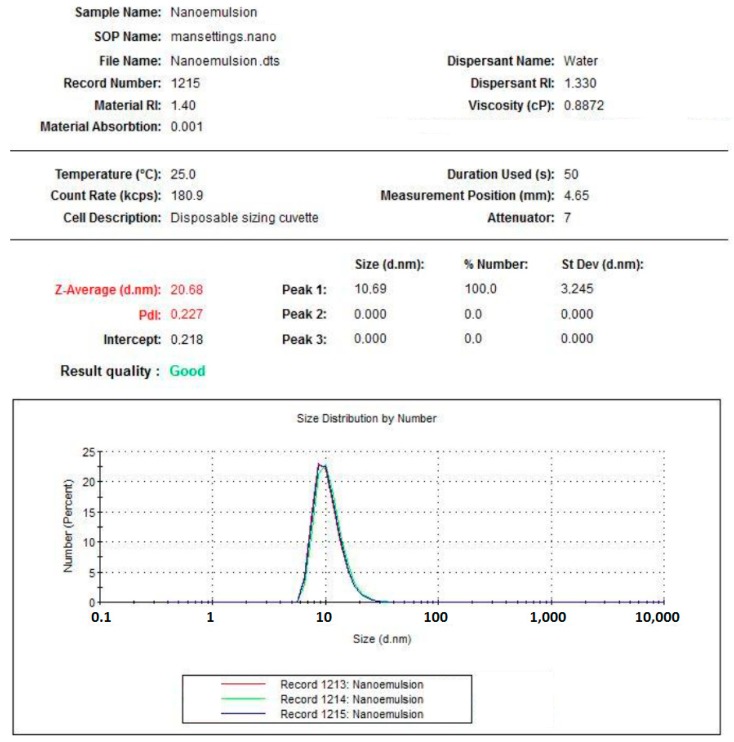
Graph plotting the size distribution of astaxanthin nanoemulsion by number.

**Figure 7:**
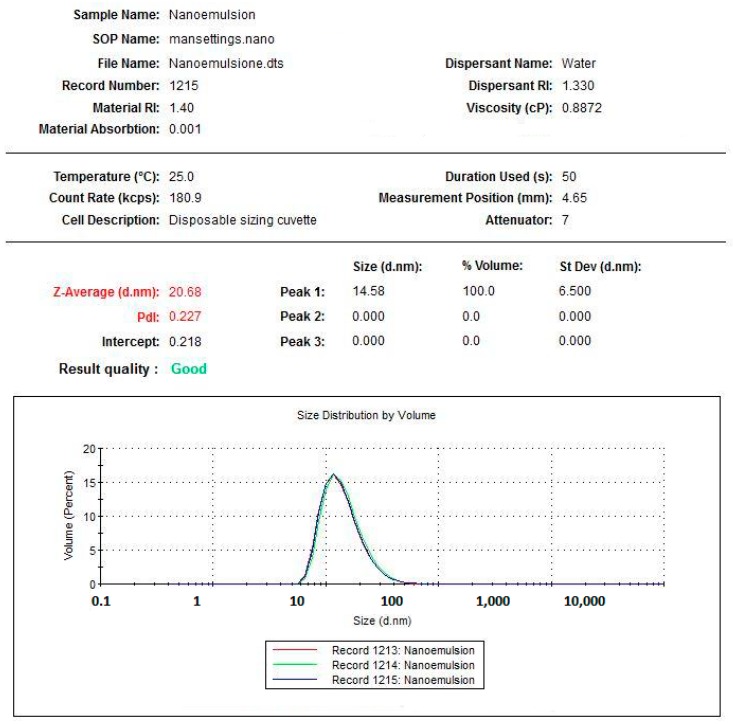
Graph plotting the size distribution of astaxanthin nanoemulsion by volume.

Figure 5, Figure 6 and Figure 7 clearly show that astaxanthin containing nanoemulsion is characterized by a single, narrow, well-shaped pick, both for the measures by number and volume weighting. The average diameter of the oily droplets is around 20 nm (z-average d.nm). The PDI of 0.2 indicates a low poly-dispersion profile with a quite uniform dispersion of the droplets. Under physical point of view, considering the average dimension of the particles and according to the published papers (refer to the section Supplementary File), this system can be considered “border-line” between a nanoemulsion and a microemulsion. Nanoemulsion, indeed, is defined as a clear kinetically stable and thermodynamically unstable liquid system, with average particles size ranging from 100 to 200 nm and a microemulsion is defined as a clear, bicontinous, kinetically and thermodynamically stable liquid system with average particle sizes lower than 50 nm. Thanks to these evidences, it is possible to argue that this system can be particularly capable of enhancing superficial surface of contact between the active ingredient entrapped in the dispersed oily phase and epithelium, in this case, lingual epithelium. Since astaxanthin is a lipophilic molecule easily soluble in oils and insoluble in water, nano-encapsulation can be hugely advantageous for efficient delivery through the lingual epithelium.

### 3.2. Titration of Astaxanthin in Raw Material and Nanoemulsion

**Figure 8:**
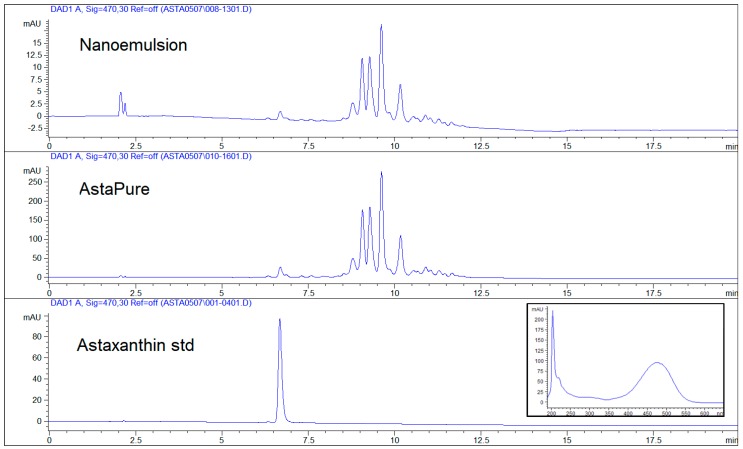
Titration of astaxanthin and correlated molecules from Astapure^TM^ (raw material), nanoemulsion and astaxanthin (analytical standard).

Figure 8 confirms the presence of astaxanthin in both Astapure^TM^ raw material and nanoemulsion with the characteristic pick at 6, 7 min. The analysis of the graph also shows a group of picks (8, 5–12 min) indicating products derived from or analogues of astaxanthin characterized by the same UV spectrum with peculiar absorption pick at 470 nm. According to Ranga et al. (2009), the mentioned picks (8–12 min) recognized in the chromatogram obtained with HPLC-DAD are mainly ascribable to astaxanthin mono and diesters [36], present in Astapure^TM^, an extract derived from *Haematococcus pluvialis* that is indeed characterized by the high content of astaxanthin in the form of fatty acid esters with the predominant presence of monoester, about 70% *w*/*w* [36], that very likely corresponds to the highest pick at about 9 min in chromatogram 1. As further confirmation, the analytical facts data sheet of Astapure^TM^ reports the presence of natural astaxanthin complex in addition to other free carotenoids such as lutein and zeaxanthin. From the titration, it was confirmed that nanoemulsion contains 0.15% *w*/*w* of astaxanthin, according to the 1.5% *w*/*w* amount of Astapure^TM^ titrated at 10% *w*/*w* of astaxanthin complex, loaded during the preparation of the nanoemulsion (Table 2).

This data, apart from the confirmation of the correct content of astaxanthin in the nanoemulsion, gives account of the stability of astaxanthin during nanoemulsion preparation: Astaxanthin is notoriously a very unstable molecule [37] and can be promptly and massively degraded when subjected to heat and UV exposure. The measures of dark room and low thermal energy chosen during the phases of preparation of the nanoemulsion allowed the full recovery of astaxanthin into the final system as the excellent data of encapsulation efficiency E*_e_* confirms (Table 3). 

Moreover, it can be argued that astaxanthin can be stabilized throughout the nanoemulsion preparation process and over time by ascorbyl palmitate (ASP), that is notoriously a lipidic antioxidant that can protect isoprenoid structure of astaxanthin and prevent it from being oxidized. 

### 3.3. Titration of Astaxanthin in Permeation Specimens

**Figure 9:**
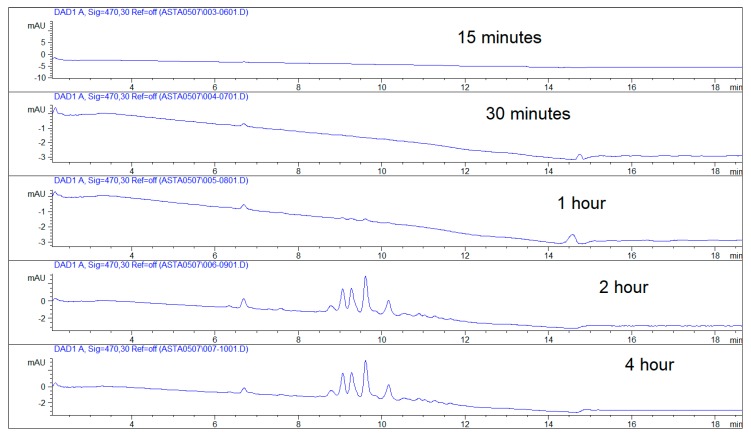
HPLC-DAD chromatograms of astaxanthin and related products in the permeated specimens over time.

**Figure 10:**
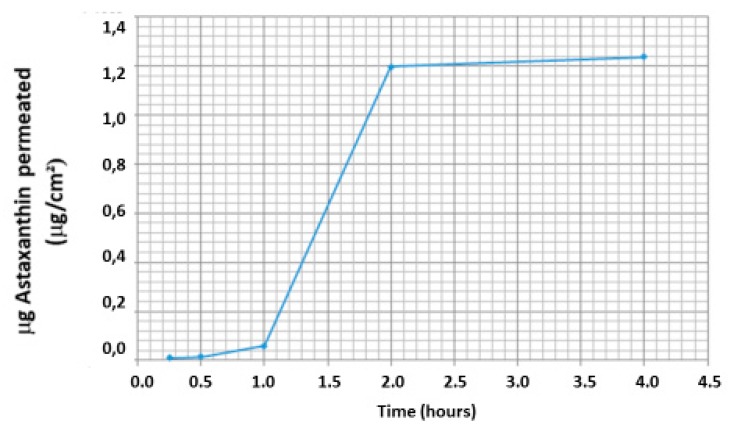
Graph of the permeation profile of astaxanthin (nanoemulsion) over time.

According to Figure 9 and Figure 10 in which the permeation rate of astaxanthin and its derivatives is represented through the HPLC-DAD titration on the five permeates specimens, as reported in Section 2.6 and Section 3.2, it is possible to recognize the peculiar astaxanthin pick in every sample. Increasing concentration of astaxanthin and its derivatives was registered in specimens over time (Table 4), indicating that astaxanthin and its derivatives accumulated in the permeated receptor liquid reaching the plateau at 2 h. From 1 h specimen to 2 h specimen, the concentration of astaxanthin and its derivatives shows a 21-fold increase (Table 4). Flux (*J_ss_*) and Apparent Permeability (*P_e_*) of astaxanthin from the nanoemulsion are equal to 6.27 ± 0.022 μm/cm^2^/h and 41.55 ± 0.442 cm/h respectively; the total amount of astaxanthin permeated after 4 h is equal to 23.6% (25.1 ± 0.24 mcg/cm^2^) of the total amount loaded in the donor compartment entrapped in the nanoemulsion (189 mcg). The amount of astaxanthin retained in the lingual tissue was not determined.

Comparing this data with that collected from Odeberg et al. [23] (Wagner–Nelson method), assessing astaxanthin bioavailability in humans from different formulations assumed orally containing 40 mg of the carotenoid with or without absorption enhancers (PS 80, Glycerol mono and dioleate, SPAN 80), it is possible to recognize comparable data of percentage of absorption over time (4 h) ranging from 4% (formulation without absorption enhancers) to 34% (PS 80 + SPAN 80). Despite this, the data are far from being completely comparable given the differences in experimental design and approach, model and the route of administration, the high hydrophilic–lipophilic balance surfactants such as PS 80, and the technological approaches capable of enhancing astaxanthin hydro-dispersibility that improve astaxanthin bioavailability seem to be confirmed.

## 4. Conclusions

Astaxanthin is a carotenoid that attracts the attention of clinicians for its well ascertained potential therapeutic activity, especially in the fields of ocular and skin health and cardiovascular disease prevention. Despite this clinical potential, astaxanthin bioavailability and stability are low and limited. Since astaxanthin is a very lipophilic molecule, the only way to deliver it in a water-based liquid system is to create an emulsion. According to this concept, liquid nanoemulsion seems to be a promising technical system for releasing astaxanthin through the lingual epithelium.

The data collected in this work show for the first time that astaxanthin can be delivered in a nanoemulsion through the lingual district, opening an interesting path to improving its poor bioavailability when assumed by the oral route. In particular, the nanoemulsion herewith described, is characterized by a good uniformity of dispersion, very low dimension of the oily droplets (around 20 nm), close to those of a microemulsion, and overall can be considered an effective and innovative pharmaceutical form for entrapping astaxanthin as the encapsulation efficiency data confirms. It is also important to emphasize that the peculiar association of surfacing agents employed to achieve this system, with specific reference to ASP, provides an overall good stability of astaxanthin during the process of nanoemulsion fabrication. This fact is of particular significance in consideration of the notorious instability of the molecule that prevents its full clinical usage. From the data collected regarding astaxanthin permeation behavior, it is possible to argue that astaxanthin reaches a pseudo-linear permeation trend during the second hour and then reaches a plateau, confirming that a quasi-steady state diffusion can be described. Taken together, these data confirm that a novel pharmaceutical form projected to deliver astaxanthin through the sublingual route has been achieved and characterized, and thanks to this peculiar form, this route can be considered, even though preliminarily, a potential effective alternative to enhance the bioavailability of this carotenoid, potentially improving its therapeutic potential. A confirmation of these preliminary data should be achieved with a pharmacological assessment of the kinetic profile of astaxanthin-containing nanoemulsion through the sublingual route in healthy human volunteers. 

## Figures and Tables

**Table 1:** Nanoemulsion qualitative composition.

Component	% *w*/*w*
Caprylic/Capric triglycerides	4.2
Polysorbate 80	2.6
Ascorbyl Palmitate	1.3
Glycerine	2.0
Deionized water	Up to 100 g
Astapure^TM^	0.15% w/2 Astaxanthin

**Table 2:** Titration of astaxanthin in Astapure^TM^ raw material (theoretical 10% *w*/*w* astaxanthin) and nanoemulsion. Results are expressed as mean ± SEM.

Sample	*w*/*w* as Total Astaxanthin
Astapure^TM^	10.04 ± 0.09
Nanoemulsion	0.151 ± 0.006

**Table 3:** E*_e_* of Astaxanthin in the nanoemulsion.

**Astapure^TM^ loaded**	1.5 g/100 mL
C*_Ast_* in nanoemulsion	0.15 gt/100 mL
Nanoemulsion E_e_ (%)	100

**Table 4:** Concentration of astaxanthin (nanoemulsion) permeated expressed as μg/mL and μg/cm^2^. Results are expressed as mean ± SEM.

Specimen	μg/mL Astaxanthin and Derivates	μg/cm^2^ Astaxanthin and Derivates
Permeated 15 min	0.019 ± 0.001	0.0108 ± 0.001
Permeated 30 min	0.025 ± 0.004	0.0142 ± 0.001
Permeated 1 h	0.102 ± 0.023	0.0577 ± 0.008
Permeated 2 h	2.116 ± 0.002	1.1980 ± 0.012
Permeated 4 h	2.184 ± 0.073	1.2365 ± 0.009

## Supplementary Materials

The following are available online at https://www.mdpi.com/1660-3397/17/9/508/s1, Text S1 Thermodynamic topics of nanoemulsions and microemulsions; Text S2. Synergy between Ascorbyl Palmitate and Polysorbate in the production of nanoemulsion.
