# Supplementary material for: Sublingual Delivery of Astaxanthin through a Novel Ascorbyl Palmitate-Based Nanoemulsion: Preliminary Data
Source: Mar Drugs. 2019 Aug 29;17(9):508. doi: 10.3390/md17090508 (PMC6780925; doi:10.3390/md17090508)
Supplement: Supplementary file 1 [file marinedrugs-17-00508-s001.pdf]

Supplementary file

5. Thermodynamic Topics of Nanoemulsions and Microemulsions

Nanoemulsions and microemulsions are physical systems in which two immiscible phases intimately dispersed one inside the other generate a nanometric droplets dispersion. Under a thermodynamic point of view the main difference between nanoemulsion and microemulsion is that the first one can be considered a kinetically stable physical form but thermodynamically metastable according to Gibbs law (Eqn.1) and the second one a kinetically and thermodynamically stable form. The main reason of this difference is that in a microemulsion, thanks to the highly efficient synergy and high amount of the emulsifying agents, micellar surfaces becomes elastic and capable of better adapting itself to the diameter reduction, in opposition to the internal droplet high pressure, even though a very low energy is imparted to the system. This fact can be explained according to Gibbs law (Eqn. 1) [1,2,3] with the occurring of a very high entropic state achieved after small droplets dispersion in the medium that balances the enthalpy energy rate of the system. The physical consequence is the reaching of a thermodynamic balance or in some cases a spontaneous emulsification process with negative values of G (Eqn. 1):

G_f = A-T S (1)

Where Gf is the free energy of formation, is the surface tension of the oil–water interface, A represents the dimensional change of interfacial micellar area, S is the variation of entropy of the system, which occurs in concomitance of the chaotic dispersion of the oily droplets into the external phase, and T is the temperature. From the afore mentioned argumentations it is possible to argue that when a nanoemulsion or microemulsion is achieved, the overall variation of A is very large because of the large number of very small droplets formed [4,5].

5.1. Synergy between Ascorbyl Palmitate and Polysorbate in the Production of Nanoemulsion

In this paper it is described a peculiar nanoemulsion system characterized by an association of surfactants capable of creating nanoemulsions according to PIT low energy method.

These technological systems can enhance bioavailability of several pharmaceutical and especially nutraceutical molecules poorly absorbed through intestinal mucosa or massively metabolized from liver microsomes so reducing their biological activity. The main innovation of this technical system consists in the peculiar association of emulsifying agents employed to reduce micellar dimension under the 100 nm, notoriously the dimensional limit under which a real nanoemulsion is recognizable even though several authors claims that an emulsion is nanosized under 500 nm [6]. To create a nanoemulsion with low energy method, it is necessary to use a binary or ternary emulsifying system in which the synergy of the surfactants allows to reduce efficiently the interfacial tension between the continuous water phase and the oily droplets. The synergy of the surfactants allows to making the micellar surface more flexible and capable of overcoming the very high interface energy produced when a droplet of internal phase is forced to reduce its dimension according to La Place law (Eqn. 2).

La Place equation:

P =2 /r (2)

where P is the gradient of pressure between out and inside the micelle; is the surface tension, and r is the radius of the micelle assuming it as spherical.

It is easy to realize that the smaller is the radius of the micelle to achieve the higher become the surface tension to win and so the energy to apply to reduce it.

As to reduce micellar diameter, it is necessary to reduce the high difference between internal and external pressure, the main strategies to achieve this goal are both the following:

• Applying a very high force on the external of the micellar surface so to reduce the ratio between internal and external micellar pressure (high pressure emulsification device), according to (eqns 2 and 3);

• Reducing the ration between 2 and r (effective surfactants association), according to (eqn. 2).

W = P ext * V (3)

Where W is the energy to oppose to the internal pressure to reduce micellar volume; Pext is the pressure to apply; and V is the volume reduction to achieve assuming that the micellar geometry is a sphere.

The difference between internal and external pressure of the droplet is the ratio between 2 and r (La Place Law).

This paper describes the use of Ascorbyl Palmitate (ASP), a notorious lipophilic ester of l-ascorbic acid obtained from the condensation of l-ascorbic acid and palmitic acid (Figure S1), as co-emulsifying agent to produce stable nanoemulsion in association with Polysorbate 80 (PS 80) [7] to deliver Astaxanthin. ASP is a well-known excipient in pharmaceutical, cosmetic and nutritional industries, and it is widely employed as antioxidant agent and molecule capable of slowing down unsaturated lipids peroxidation.

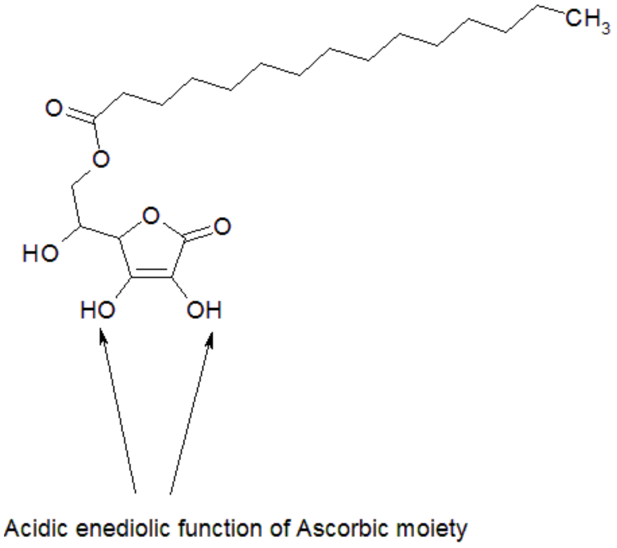

Figure S1. l-Ascorbyl Palmitate structure.

Thanks to the palmitic radical, in fact, ASP can be easily solved in both solid and liquid lipid/fat-based preparation and work to reduce oxidation and peroxidation in force of the effective reducing ascorbic radical. Some papers describe also its use as lipophilic form of l-ascorbic acid potentially capable of improving skin bioavailability of l-ascorbic acid [8]. Thanks to its amphiphilic structure conferred by hydrophilic ascorbic moiety and lipophilic palmitic chain, ASP perfectly complies with the definition of surfactant. There are, indeed, several papers describing the chemical-physical behavior of ASP previously ionized in alkaline water to self-assembly and form micellar structures called ASPASOMES [9] (Figure S2), investigated for the capability to form vesicles potentially involved in the improvement of some drugs bioavailability.

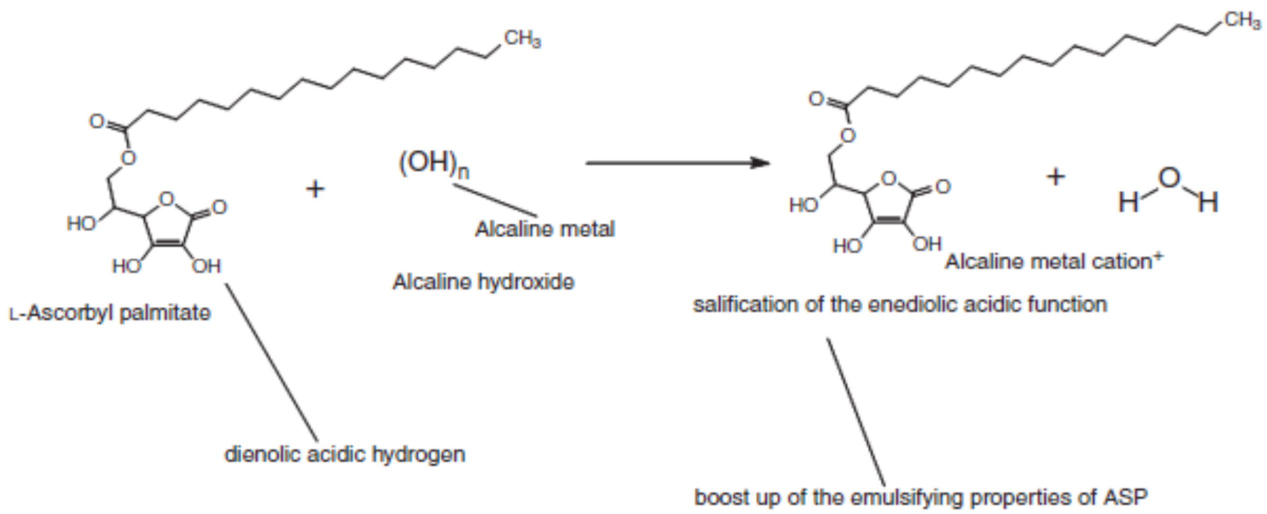

Figure S2. Salification of ascorbyl palmitate by the side of alkali.

Synergy between ASP and PS 80 seems to find works in reducing droplet surface tension, thanks to a regular geometric disposition of the surfactant molecules taking place to an elastic monolayer surrounding oily droplet surface (Figure S3).

Particularly, the high structure affinity between carbon chains of both emulsifiers and triglycerides could promotes molecules packing up. On the other hand, we could speculate that double bond in the center of PS 80 carbon chain (Figure S4) could improve flexibility and elasticity of the oily droplet surface, so promoting an easier diameter reduction in the micelle.

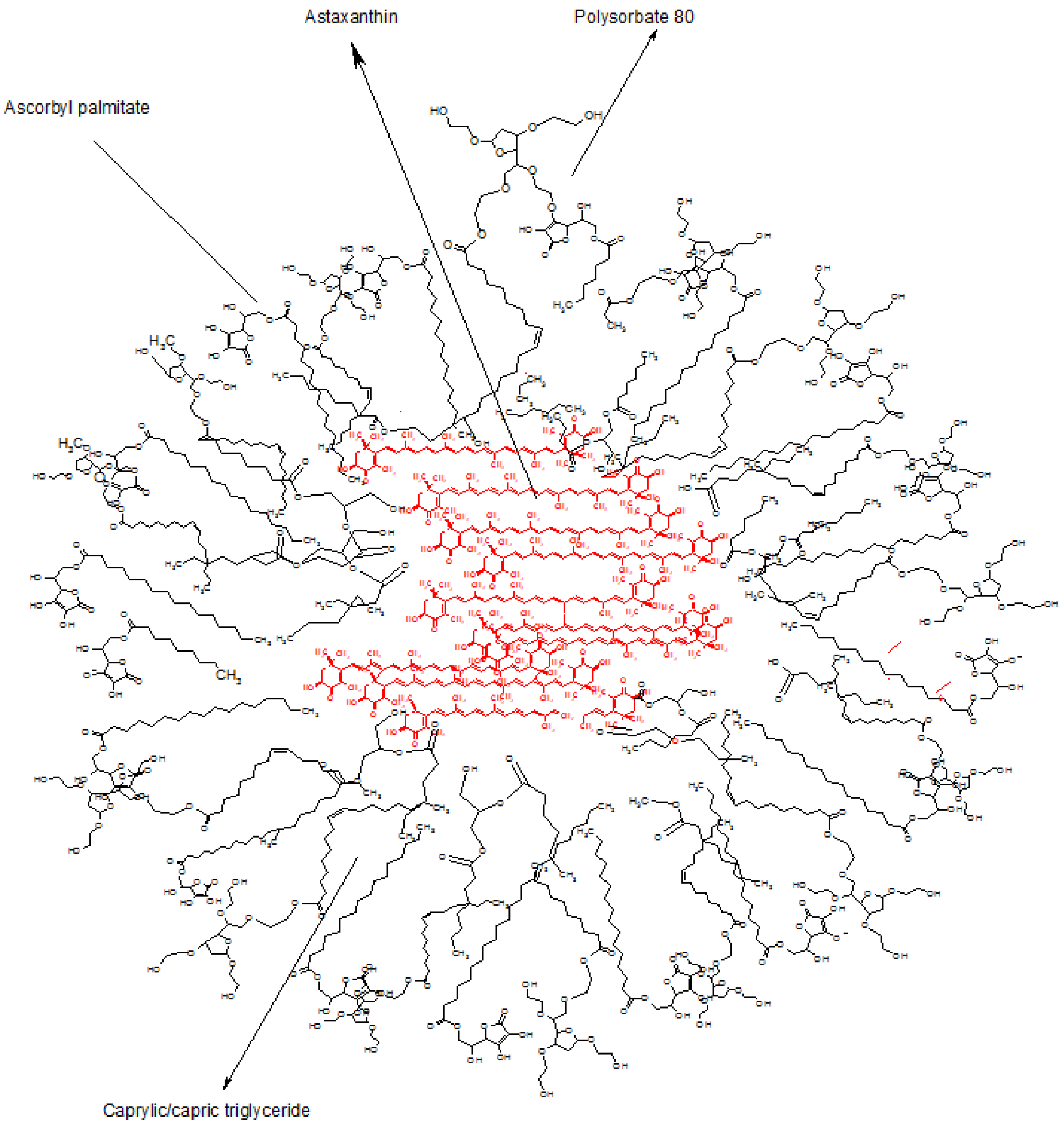

Figure S3. Theoretical structure of an oily nanodroplet of MCT (Caprylic/Capric Triglyceride) in which Astaxanthin is solubilized, surrounded by a monolayer of PS 80 and ASP.

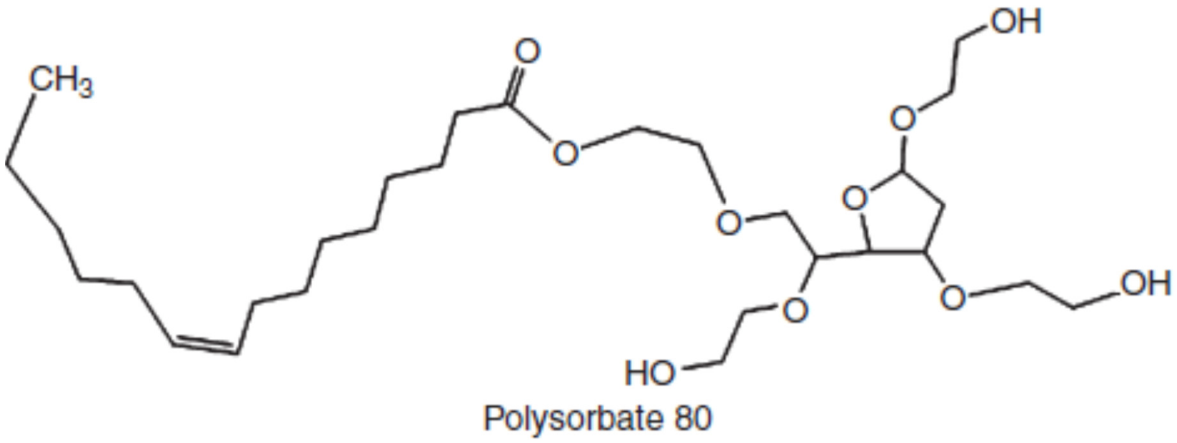

Figure S4. Structure of polysorbate 80.

Bibliography

1. El-Aasser, M.S.; Lack, C.D.; Vanderhoff, J.W.; Fowkes, F.M. Miniemulsification process: different form of spontaneous emulsification. *Colloid Surf.* **1988**, *29*, 103.
2. Nakajima, H. Industrial Applications of Microemulsions. Marcel Dekker, New York, 1997.
3. Fernandez, P.; Andre¢, V.; Rieger, J.; Kuhnle, A. Nano-emulsion formation by emulsion phase inversion. Colloids Surf. *A Physicochem. Eng. Asp*. **2004**, *251*, 53–58.
4. Solans, C.; Esquena, J. ; Forgiarini, A.M. Absorption and Aggregation of Surfactants in Solution, Vol. 109. Marcel Dekker, New York, 2003.
5. Miller, C.A.; Neogi, P. Thermodynamics of microemulsions: combined effects of dispersion entropy of drops and bending energy of surfactant films. *AIChE J*. **1980**, *26*, 212–220.
6. Bouchemal, K.; Briancon, S.; Perrier, E.; Fessi, H. Nano-emulsion formulation using spontaneous emulsification: solvent, oil and surfactant optimization. *Int J Clin Pharm*. **2004**, *280,* 241–251.
7. Fratter, A., Semenzato, A. New association of surfactants for the production of food and cosmetic nanoemulsions: preliminary development and characterization. *Int J Cosmet Sci.* **2011**;*33*, 443–449.
8. Pinnell, S.R.; Yang, H.; Omar, M.; Monteiro-Riviere, N.; DeBuys, H.V.; Walker, L.C.; Wang, Y.; Levine, M. Topical L-ascorbic acid: percutaneous absorption studies. *Dermatol Surg*. **2001**, 27, 137–142.
9. Gopinath, D.; Ravi, D.; Rao, B.R.; Apte, S.S.; Renuka, D.; Rambhau, D. Ascorbyl palmitate vesicles (Aspasomes): formation, characterization and applications. *Int J Pharm*. **2004**, *271*, 95–113.
